# Local environmental variables are key drivers of ant taxonomic and functional beta-diversity in a Mediterranean dryland

**DOI:** 10.1038/s41598-021-82059-w

**Published:** 2021-01-27

**Authors:** Clara Frasconi Wendt, Ana Ceia-Hasse, Alice Nunes, Robin Verble, Giacomo Santini, Mário Boieiro, Cristina Branquinho

**Affiliations:** 1 cE3c-Centre for Ecology, Evolution and Environmental Changes, Faculty of Sciences, University of Lisbon, Campo Grande, C2, 1749-016 Lisbon, Portugal; 2 Department of Biology, University of Florence, Via Madonna del Piano 6, 50019 Sesto Fiorentino, Italy; 3 Institute of Hygiene and Tropical Medicine, NOVA University of Lisbon, Rua da Junqueira 100, 1349-008 Lisbon, Portugal; 4 Department of Biological Sciences, Missouri University of Science and Technology, Rolla, MO USA; 5 cE3c-Centre for Ecology, Evolution and Environmental Changes/Azorean Biodiversity Group, University of the Azores, Angra do Heroísmo, 9700-042 Terceira, Azores Portugal

**Keywords:** Ecology, Ecology

## Abstract

The decomposition of beta-diversity (β-diversity) into its replacement (β_repl_) and richness (β_rich_) components in combination with a taxonomic and functional approach, may help to identify processes driving community composition along environmental gradients. We aimed to understand which abiotic and spatial variables influence ant β-diversity and identify which processes may drive ant β-diversity patterns in Mediterranean drylands by measuring the percentage of variation in ant taxonomic and functional β-diversity explained by local environmental, regional climatic and spatial variables. We found that taxonomic and functional replacement (β_repl_) primarily drove patterns in overall β-diversity (β_tot_). Variation partitioning analysis showed that respectively 16.8%, 12.9% and 21.6% of taxonomic β_tot_, β_repl_ and β_rich_ variation were mainly explained by local environmental variables. Local environmental variables were also the main determinants of functional β-diversity, explaining 20.4%, 17.9% and 23.2% of β_tot_, β_repl_ and β_rich_ variation, respectively. Findings suggest that niche-based processes drive changes in ant β-diversity, as local environmental variables may act as environmental filters on species and trait composition. While we found that local environmental variables were important predictors of ant β-diversity, further analysis should address the contribution of other mechanisms, e.g. competitive exclusion and resource partitioning, on ant β-diversity.

## Introduction

Measurements of biological diversity and its responses to environmental changes are key issues in ecology^1^. To asses changes in diversity across different communities along climatic gradients, researchers rely on beta diversity (β-diversity), which is defined as the difference in species composition between two or more communities^2^. Recently, a framework was developed to assess the contribution of the two components of total β-diversity (differences in species richness and composition between sites), namely: (1) species replacement (i.e. differences in diversity due to species replacement) and (2) species richness differences (i.e. differences in numbers of species present)^3,4^. However, taxonomic β-diversity alone may not fully elucidate the underlying processes regulating community assemblages^5^. To overcome this issue, a functional trait approach has been applied to the β-diversity concept (e.g.^1,4–6^). The functional approach to β-diversity by Carvalho et al.^3^ follows a similar rationale to the one used in the taxonomical approach and total functional β-diversity (β_tot_) can be decomposed into two components: (1) trait value replacement (β_repl_) and, (2) trait value richness (loss/gain; β_rich_).

Using multiple approaches and exploring the components of taxonomic and functional β-diversity in tandem, allows us to more completely understand ecological mechanisms regulating diversity. Combining taxonomic and functional β-diversity increases our understanding of community patterns and their regulatory processes (i.e., neutral- *versus* niche-based), as we can see from its recent generalized application [e.g.^5–8^]. If neutral-based processes are dominant, community composition is the result of random associations of species and their functional traits. As a result, β-diversity is expected to increase as the distance between sites increases (across space) while it would remain constant across environmental gradients^9^. Niche-based processes include biotic and abiotic filters, which have similar effects on community composition and are therefore difficult to disentangle^10^. For example, under abiotic filtering, environmental conditions may exclude some species and limit some trait establishment and persistence by selecting for or against species and ecological strategies^11^. As a result, with abiotic filters, β-diversity is expected to be constant across space and increase along an environmental gradient, with communities in the same environmental conditions sharing similar traits^9,12^. In the extreme tails of environmental gradients, we expect that environmental differences match biological differences, while in areas with similar environmental conditions, low species and trait differences are expected. Instead, under similar environmental conditions, biotic filters, such as competitive exclusion, may lead to a higher dissimilarity of traits^13^.

Niche-based processes may lead to high dissimilarity in both taxonomic and functional β-diversity between two extreme ends of a gradient^6,14^; however, taxonomic and functional β-diversity may also show distinct patterns over the length of the entire environmental gradient^5,14^. Along an environmental gradient, high taxonomic dissimilarity may be coupled to low functional dissimilarity (functionally similar species), indicating the presence of species with similar combinations of traits^6^. For example, different regions sharing similar environmental conditions may have high species dissimilarity and low functional β-diversity^5^. Therefore, high functional diversity may result either from the replacement of functionally different species, indicating abiotic filtering, or from the loss/gain of functional strategies, which may be related to a different intensity of the niche-based processes^14^.

Ants represent a key group to examine functional and taxonomic diversity because they are diverse, abundant, and have an essential role as ecosystem engineers, especially in drylands where they are often associated with important ecological functions (e.g. enhancing soil properties and seed dispersal)^15,16^. Ant communities are shaped by abiotic, namely small-spatial scale (local) environmental factors, such as plant composition, vegetation structure, soil characteristics and productivity^17,18^ and large-scale (regional) climate variables, such as temperature and precipitation^19,20^ and biotic interactions, e.g. competitive exclusion. Furthermore, the role of ants in drylands together with their potential as ecological indicators^20^, makes this a key taxon to be monitored in response to environmental changes. Ant β-diversity has been assessed for different ecosystems^21^, although these studies focused on elevational gradients^22,23^. However, to our knowledge, previous studies along aridity gradients have either measured the link between aridity and ant species diversity or functional traits, but not taxonomic and functional β-diversity (e.g.^18^). Given that Mediterranean drylands are particularly vulnerable to aridity increase^24^, they offer an interesting context in which to assess the processes governing ant diversity and community assemblages along climatic environmental gradients.

Our goals were to analyze how ant taxonomic and functional β-diversity change along climatic environmental gradients and to identify which components (replacement or richness differences) contribute most to overall β-diversity. Furthermore, we evaluated the contribution of local environmental, regional climatic and spatial variables as determinants of ant β-diversity variation. To do that, we performed variation partition analysis, which has been used to disentangle the influence of the selected variables on community changes and to understand whether niche- or neutral-based processes drive β-diversity (e.g.^9^). We specifically addressed the two following questions: (1) which components (β_repl_ and β_rich_) drive ant taxonomic and functional β_tot_? and (2) which abiotic factors explain variation in taxonomic and functional β-diversity and how can they help us to infer on the ecological processes driving taxonomic and functional β-diversity along climatic environmental gradients? Based on previous studies^22,25^ we expected a higher contribution of the β_repl_, over the β_rich_ component, to ant β_tot_, and that along climatic environmental gradients, niche-based processes play the most important role in structuring ant communities.

## Results

In total, we collected 36 ant species representing three sub-families (Dolichoderinae, Formicinae and Mymicinae). The richest sub-family was Myrmicinae, which accounted for 20 species, followed by Formicinae with 12 species (Table S1 in Supplementary Information). The genus *Temnothorax* (Mayr, 1861) accounted for most species (6 species), followed by the genus *Camponotus* (Mayr 1861) (4 species) and *Aphaenogaster* (Mayr, 1853), *Messor* (Forel, 1890) and *Tapinoma* (Foerster, 1850) (all with 3 species, respectively). Five ant species (*Aphaenogaster senilis* (Mayr, 1853), *Cataglyphis hispanica* (Emery, 1906), *Formica subrufa* (Roger, 1859), *Messor barbarus* (Linnaeus, 1867) and *Temnothorax nylanderi* (Foerster, 1850) were widespread in the study area, occurring in more than 20 sites, eight species occurred in between 10 and 20 sampling sites, while most species (S = 23) were restricted to less than 10 sites.

We found that sample completeness was high and similar between sampling sites (0.78 ± 0.09, mean ± standard deviation).

The fourth-corner analysis found a marginally significant trait-environmental relationship (p = 0.09), suggesting that the traits explain some of the variation in the responses of species to environmental gradients (Figure S1). In particular, we found a strong negative association between epigeic nesting and annual mean temperature (bio1), low polymorphism and mean diurnal range (bio02), body size (Weber’s length; WL) and relative cover of woody plant species, and between diurnal and nocturnal activity and mean plant height. A slight negative association was found between head length (HL) and aridity, HL and mean normalized difference vegetation index (NDVI), nesting under stones and aridity, generalist diet and biomass, medium polymorphism and mean plant height, nocturnal activity and bare soil (%). Relative cover of woody plant species was negatively correlated with nocturnal activity and with high polymorphism. We found a positive association between seed-based diet, low polymorphism, mound presence and nocturnal activity and mean plant height, and between epigeic nesting and herbaceous biomass, suggesting that ants with those traits were more frequent in sites with more complex vegetation structure. A slight positive relationship was found between arboreal nesting and bio1, sugar-based diet and NDVI, and sugar-based diet and dry herbaceous biomass, while polymorphism was positively associated to bio2, dry herbaceous biomass and to bare soil (%).

For taxonomic β-diversity, mean pairwise dissimilarity (± standard deviation) for Tβ_tot_ was 0.616 (± 0.128), and mean Tβ_repl_ and mean Tβ_rich_ were 0.427 (± 0.197) and 0.189 (± 0.145), respectively. For functional β-diversity, mean pairwise dissimilarity (± standard deviation) for Fβ_tot_ was 0.488 (± 0.118), 0.317 (± 0.169) for Fβ_repl_ and 0.170 (± 0.123) for Fβ_rich_. When we used less traits and a different trait coding (fuzzy coding for all qualitative traits), mean pairwise dissimilarity for functional β-diversity was similar despite being slightly lower. In particular, we found that with decreasing number of ant functional traits, namely 7 and 4 traits, and using the original coding, mean pairwise dissimilarity was slightly lower for β_tot_, β_repl_ and β_rich_ (Table S2 in Supplementary Information). As for the functional β-diversity computed with 11 traits, the β_repl_ component contributed mostly to the overall functional β-diversity (β_tot_). When we measured ant functional β-diversity using 11, 7 and 4 traits and a different coding for qualitative traits (fuzzy coding), we found similar results, with Fβ_repl_ rather than Fβ_rich_ mainly contributing to overall Fβ_tot_ (Table S2 in Supplementary Information). This analysis indicated our results are robust to changes in trait numbers and in trait coding. Thus, in further analysis we used 11 traits with the original coding.

**Figure 1:**
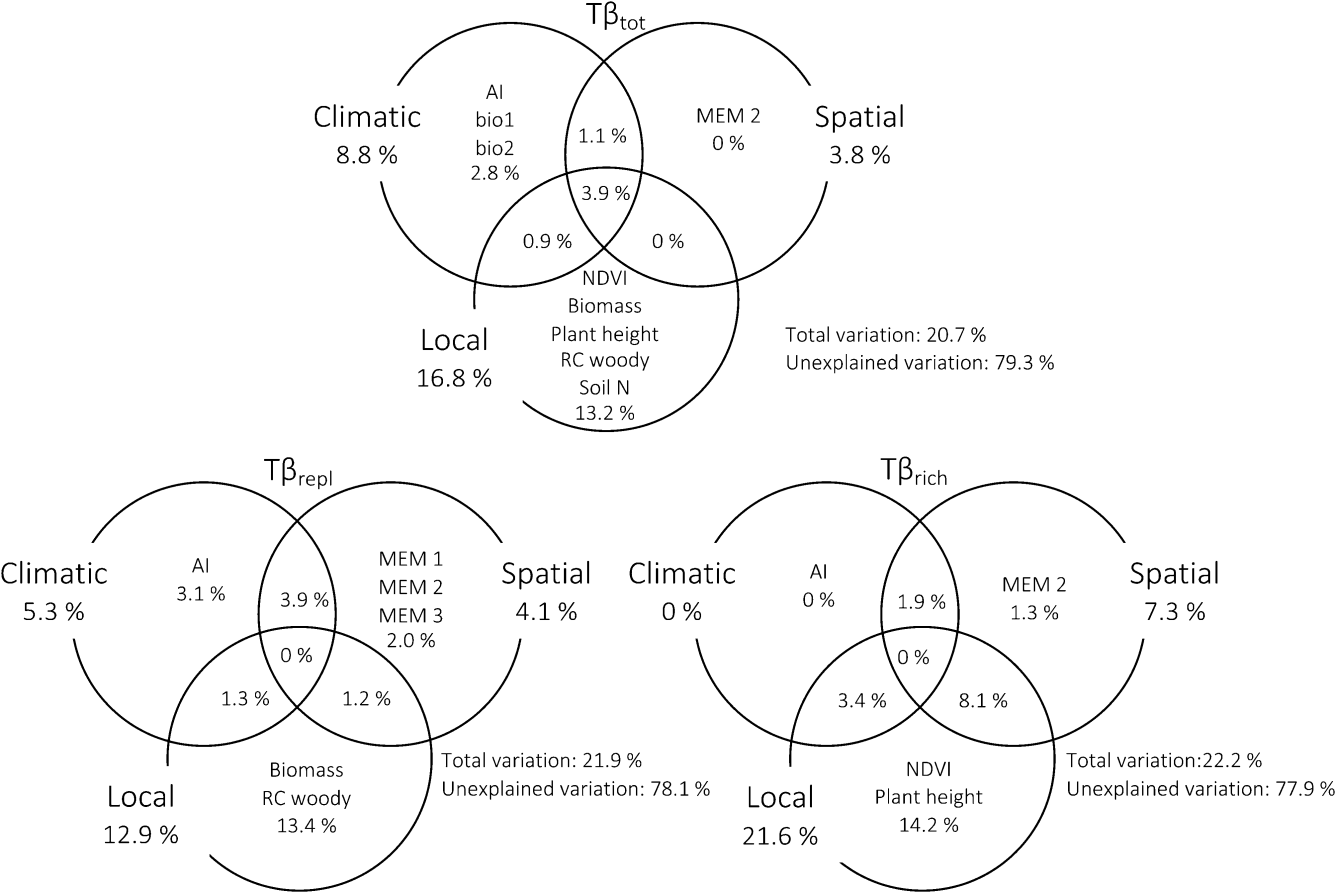
Variation partitioning of ant taxonomic beta diversity. Venn diagrams showing the groups of variables explaining variation in Tβ_tot_, Tβ_repl_ and Tβ_rich_, and the percentage of variation (adjusted R^2^) explained by each effect. Percentages inside circles indicate pure contributions and percentages within intersections indicate shared contributions. Percentages outside circles refer to the total contribution of local, climatic and spatial variables to the variation in taxonomic β-diversity. When an effect has a negative adjusted R^2^, then the sum of pure and shared effects does not equal to the total variation explained. In this case the sum is equal to the total variation explained when considering the negative value. Variable names stand for: *AI* Aridity Index, *bio1* annual mean temperature, *bio2* temperature mean diurnal range, *Biomass* dry herbaceous biomass, *NDVI* mean normalized difference vegetation index, *Plant height* mean plant height, *RC* *woody* relative cover of woody plant species, *Soil N* soil nitrogen. MEM variables correspond to the spatial relationships among sampling sites.

The Tβ_tot_ model explained 20.7% of the variation along the climatic gradient by the following factors: (1) pure local (13.2%), (2) pure climatic (2.8%) and (3) pure spatial (0%) (Fig. 1). The Tβ_repl_ model explained 21.9% of its variation along the climatic gradient by the following factors: (1) pure local (13.4%), (2) pure climatic (3.1%) and (3) pure spatial (2%). Regarding Tβ_rich_, the forward selection procedure selected local, climatic and spatial variables, which together account for 22.2% of the variation explained. Tβ_rich_ was explained by: (1) pure local (14.2%), (2) pure climatic (0%) and (3) pure spatial (1.3%) variables.

**Figure 2:**
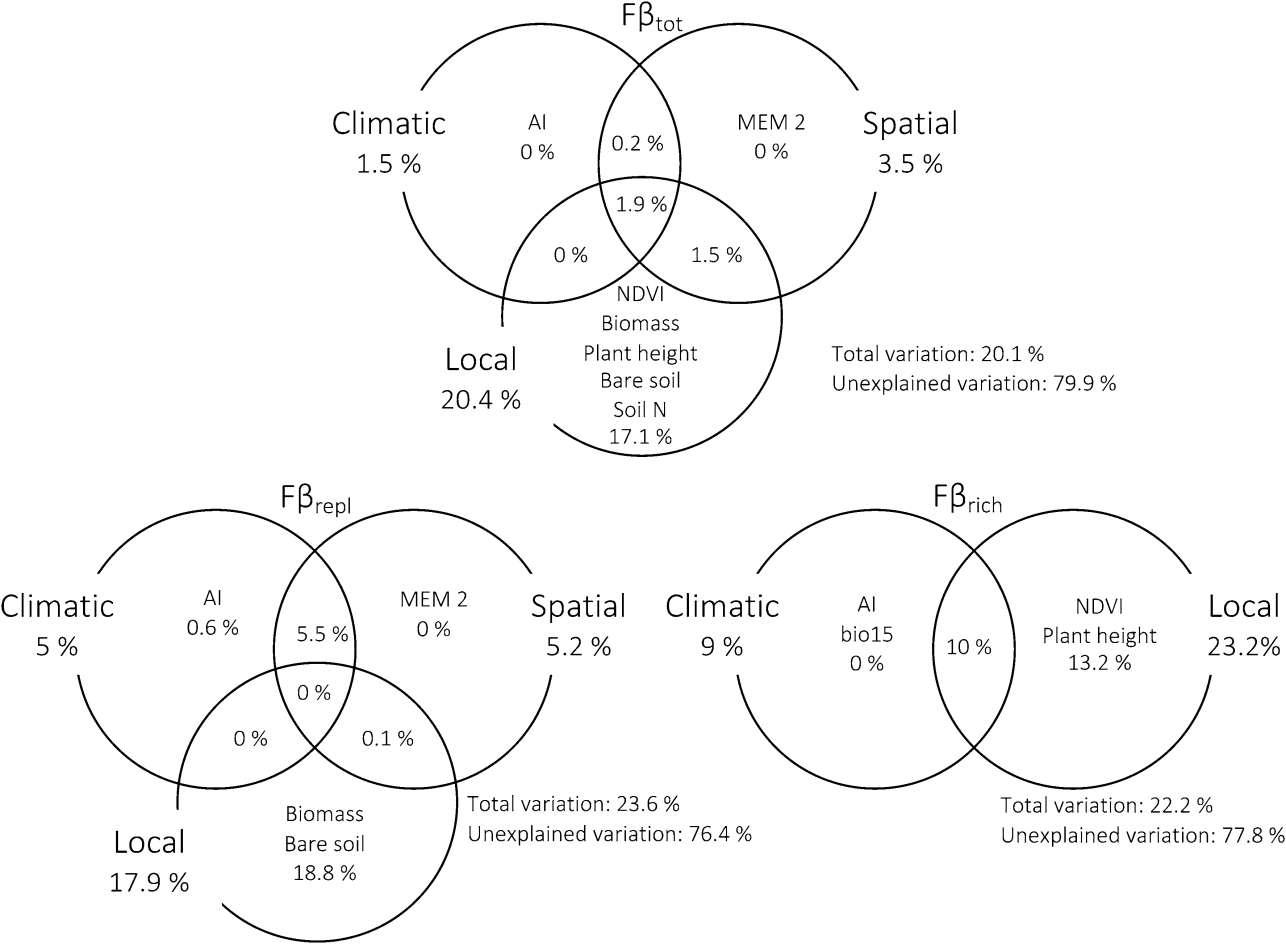
Variation partitioning of ant functional beta diversity. Venn diagrams showing the variables explaining variation in Fβ_tot_, Fβ_repl_ and Fβ_rich_, and the percentage of variation (adjusted R^2^) explained by each effect. Percentages inside circles indicate pure contributions and percentages within intersections indicate shared contributions. Percentages outside circles refer to the total contribution of local, climatic and spatial variables to the variation in functional β-diversity. When an effect has a negative adjusted R^2^, then the sum of pure and shared effects does not equal to the total variation explained. In this case the sum is equal to the total variation explained when considering the negative value. Variable names stand for: *AI* Aridity Index, *bio15* precipitation seasonality, *Biomass* dry herbaceous biomass, *NDVI* mean normalized difference vegetation index, *Plant height* mean plant height, *Bare soil* percentage of bare soil, *Soil N* soil nitrogen. MEM variables correspond to the spatial relationships among sampling sites.

Variation partitioning for Fβ_tot_ showed in general similar patterns of those found for Tβ_tot_ (Fig. 2). The model of the Fβ_tot_ explained 20.1% of its variation, with pure local, pure climatic and pure spatial factors contributing respectively with 17.1%, 0% and 0%. The component of Fβ_repl_ model explained 23.6% based on local, climatic and spatial variables, contributing respectively with 18.8%, 0.6% and 0%. The Fβ_rich_ model explained 22.2% of its variation due to local and climatic factors with 13.2% and 0%, respectively. For Tβ_rich_, the spatial component did not contribute to variation in Fβ_rich_ and was therefore also not considered for further discussion and data treatments (Fig. 2).

## Discussion

We found that both Tβ_tot_ and Fβ_tot_ were primarily driven by the species and trait value replacement components (β_repl_) respectively, which appears to be common across a wide range of taxa, including ants^17,25–27^. Species and trait value replacement components playing a major role in generating the observed ant β-diversity patterns and the trait-environment associations, suggest that functionally unique species are replaced as environmental differences between sampling sites increase. Taxonomic and functional β-diversity components shared similar sets of variables explaining β-diversity variation, e.g. vegetation structure, productivity and aridity. Furthermore, the higher contribution of the β_repl_ component to β_tot_, in both taxonomic and functional anlayses, combined with the larger contribution of local environmental and regional climatic factors as determinants of β-diversity agree with previous findings in a similar environment^28,29^ and may corroborate the hypothesis that niche-based processes drive ant β-diversity in this Mediterranean dryland.

The contribution of different environmental factors to changes in taxonomic and functional β-diversity has been shown in other important invertebrate groups as well^30^, and may indicate that different environmental variables act as abiotic filters on ant taxonomic and functional β-diversity. In particular, in drylands the role of local environmental and regional climatic variables in explaining variation in ant species and trait composition has been highlighted^25^. However, while these authors^25^ found no relationship between ant β-diversity and habitat structure (local factors, e.g. trees density and diversity), our results show that local environmental factors, such as habitat structure, productivity and soil characteristics were the major determinants structuring variation in ant β-diversity. Furthermore, we found that some ant functional traits were associated to those environmental variables and changed along the gradients. For example, ants with a seed-based diet increased in sites with a higher plant height, epigeic ants (nesting above ground) increased in sites with more herbaceous biomass, while in shrubby sites (high relative cover of woody plant species) ants with large body size decreased.

In our study, regional climatic variables explained some changes in ant taxonomic and functional β-diversity: as found by other studies^18–20,22^, aridity, temperature and precipitation seasonality shaped ant functional structure and β-diversity. However, the contribution of regional climatic variables was low and mainly through shared effects with local environmental factors, as for the Tβ_rich_ and Fβ_rich_ components, and spatial variables, e.g. Tβ_repl_ and Fβ_repl_ components. The shared effects between local environmental and regional climatic variables suggest that these two sets of factors are connected and interact with each other. As found recently along the same aridity gradient, climatic variables, such as aridity, summer precipitation and winter temperature directly influence vegetation structure and productivity^31^. Instead, the shared effects between regional climatic and spatial factors, and also local environmental and spatial factors, indicate that ant β-diversity variation is explained by spatially structured environmental and climatic variables as well, although percentages were low.

While these results narrow down the abiotic factors shaping ant beta-diversity in drylands and point to niche-based processes driving changes in ant taxonomic and functional β-diversity, identifying which ecological mechanism among the niche-based processes is responsible for the observed pattern remains difficult^10^. In concert, these results suggest that the occurrence of different environmental conditions through space may have selected species with unique functional strategies^10^, leading to taxonomically and functionally distinct communities^11^. Apart from abiotic filters, competitive exclusion and resource partitioning may drive ant beta diversity patterns too^9,10^. However, quantifying and disentangling the contribution of these different filters on β-diversity brings many challenges, as they often covary and create very similar patterns in biodiversity^10^. Furthermore, measuring competitive exclusion in the field is quite difficult (e.g.^32^) although it should be addressed in a future study. Thus, we acknowledge that abiotic and biotic filters may act synergistically on ant β-diversity in drylands, and while we found that local environmental variables were associated to some ant functional traits and explained some of the variation in ant β-diversity, we cannot exclude that part of the patterns observed may be a result of resource partitioning and competitive exclusion too^10^.

Spatial variables also explained some of the variation in ant taxonomic and functional β-diversity, although for all components the contribution was low. Similar to another study^33^, our findings emphasize that neutral-based processes play only a secondary role in shaping ant β-diversity. We provide two explanations for the observed spatial effect on ant β-diversity in our study gradient. First, the spatial component may represent biotic interactions at local scale and other unmeasured variables that show a spatial distribution, as environmental variables are often spatially structured^34,35^. Second, the influence of spatial variables on β-diversity suggests that neutral-based processes may also have an influence on ant β-diversity in drylands. Neutral-based processes drive diversity when a community is primarily influenced by the neighbouring community and the dispersal rate of species in its immediate surroundings (dispersal limitations)^35^. Neutral-based processes are usually found to be stronger at small spatial scales, given that biotic conditions may change quickly at small-scales, while as the spatial scale increases, environmental differences accumulate, matching a shift in biotic conditions^36^.

Lastly, we would like to stress out three important aspects of this study. First, our analysis revealed that the local, climatic and spatial variables only explained a low percentage of the total variation and just identified some of the drivers shaping ant taxonomic and functional β-diversity in drylands. Second, we acknowledge that the approach we follow^3,4^, while bringing many advantages, also comes with some pitfalls^8^. Functional β-diversity based on Carvalho’s et al.^3^ approach allows to distinguish between differences in assemblages due to true replacement of functional traits or due to loss/gains of functional traits, and neither species replacement is overestimated nor species richness is underestimated^4^. However, Cardoso’s et al.^4^ method produces a functional space with a lower quality, as the functional distances between species generated from the functional dendrogram^37^ seem to differ more from the initial dissimilarity matrix than those created in a functional ordination^38^, and the two functional β-diversity components seem not to be independent from one another^8^. Third, the present study had temporal and spatial limitations, specifically a low temporal span with sampling being limited to one of the yearly peak of ant activity and a short length of the gradient, restricted to southwestern Iberia and including only two aridity classes. An expansion of the gradient in space and time, e.g. through the inclusion of additional aridity classes and repeated sampling over more years, might be needed to reinforce our findings.

Concluding, in the present study, we provide evidence that environmental variables explain part of ant β-diversity in Mediterranean drylands, which is mostly driven by species and trait replacement. Regarding local environmental and regional climatic variables, this study supports previous studies (e.g.^29^) addressing the need to include abiotic explanatory variables acting at different scales, and biotic factors to assess changes in ant β-diversity. Moreover, we highlighted the importance of combining multiple diversity approaches to understand changes in ant β-diversity. Based on our results, we suggested that niche-based processes, including abiotic and biotic filters, may shape ant β-diversity in Mediterranean drylands. This result is timely and important, given that we need to improve our understanding of ant diversity patterns in drylands^39^ and that ants more so than other key groups are predicted to respond in a highly sensitive way to increased aridity and higher temperatures^40^. Indeed, the expansion of anthropogenic influence and climatic changes in dryland area, as well as changes in temperature and precipitation amount and patterns in the Mediterranean region^24,41^ may lead to a rearrangement of the environmental factors influencing ant β-diversity, which may, in turn, accelerate the observed ant species and trait value replacement, or even shift the relative contribution of each component to the total β-diversity.

## Materials and methods

### Study area

This study was carried out along an aridity gradient in the drylands of the southwestern Iberian Peninsula. The study area is a low density holm oak (*Quercus ilex*) woodland known as the Montado. The site understory is characterized by shrubs of the genera, *Cistus* and *Lavandula* (among others). The Montado supports sustainable anthropogenic activities, such as low-intensity grazing and cork harvest, in addition to woodland biodiversity^42^.

Our sampling design was composed of a total of 30 sampling sites and was stratified to the aridity index (AI)^43^, which is defined by the United Nations as the ratio between the mean annual precipitation over the annual potential evapotranspiration (Figure S2 in Supplementary Information). We extracted the AI values for each sampling site from the global aridity database (https://cgiarcsi.community/data/global-aridity-and-pet-database)^44^. Along the aridity gradient, high levels of AI equate to low aridity, while low levels of AI represent high aridity. The sampling area includes semi-arid to dry sub-humid aridity classes with AI ranging between 0.42 and 0.54. The vegetation of the sampling sites has been the subject of past studies (e.g.^31,45^) and was unimpacted by common local environmental disturbances such as heavy grazing, recent agricultural activities and past fires.

### Sampling

#### Ant sampling and functional traits

Ants were sampled between May and June 2017, which coincides with one of the peaks in ant activity during the year^46^. We deployed 10 pitfall traps per sampling site, which consisted of 50 ml Falcon tubes, filled with 10% diluited ethylene glycol and a few drops of liquid detergent to reduce surface tension. Pitfall traps were arranged in a circle, with a diameter of 10 m. Pitfall traps were spaced at 5 m apart each along the circumference of the circle and left in the field for 5 days. Samples were collected and transported to the laboratory, where ants were sorted and identified to species^47^ using a stereomicroscope.

Traits were selected according to their ecological importance (Table S3 in Supplementary Information), and included continuous, categorical, ordinal and binary traits. Continuous traits include ant Weber’s length, which corresponds to the length of the ant mesosoma in profile; head length and femur length. Continuous trait values were obtained by measuring 15 individuals per species under a stereomicroscope and taking the mean value for each species. For less abundant species (N < 15), we measured all available individuals. Categorical traits included ant diet preferences, activity period, and nesting sites. We measured one ordinal trait, polymorphism, which measures the degree of differences in worker size within the same species. We also selected four binary traits: ant behaviour, ant color, mound presence, and foraging strategy. Categorical traits were retrieved from the available literature and the online database *GlobalAnts* (http://globalants.org)^20,48^.

#### Environmental variables

Several environmental variables were selected based on their presumed influence on ant traits and species community composition (e.g.^19,20^). Environmental and climatic variables were divided into two categories: local environmental factors, which included variables measured directly in the field at a scale of < 50 m, and regional climatic factors, which included variables retrieved from the global aridity database^44^ and the WorldClim database^49^ at a scale of > 1000 m.

Local environmental factors that are usually associated with changes in ant diversity and distribution were collected at each sampling site and included: mean plant height, dry herbaceous biomass, soil nitrogen and soil carbon:nitrogen ratio, plant species richness, the relative cover of woody species and normalized differenced vegetation index (NDVI). Mean plant height, dry herbaceous biomass, species richness, and relative cover of woody plant species describe habitat structure and environmental heterogeneity, whereas the NDVI is used as a proxy for vegetation productivity^50^. Soil characteristics inform on the rugosity of the environment and usually are a key driver of ant morphological traits^51^ since ground-dwelling ants move mainly between the soil–plant interface.

Along a 20 m transect, we measured maximum plant height and soil cover type every 50 cm, by holding a rod perpendicular to the soil surface and recording maximum plant height and soil cover touching the rod (Table S4 in Supplementary Information). For each sampling site, we used the average plant height per site and recorded the percentage of the soil surface cover type (bare soil, leaf litter, mosses). At each sampling site during the same period as for ant sampling, we collected three replicates of herbaceous biomass (from quadrats of 0.50 × 0.50 m) and three soil sub-samples, which were later combined into a composite sample. Samples of herbaceous biomass were dried (for three days at 60 °C) and weigted to obtain mean dry herbaceous biomass per area for each sampling site (Table S4 in Supplementary Information). Soil nitrogen and the carbon:nitrogen ratios were obtained for each sampling site (Table S4 in Supplementary Information).

For each site, we utilized previously recorded measurements of plant species richness and the relative cover of woody species (Table S4 in Supplementary Information)^45^, which were measured in the field using the point-intercept method. Values for NDVI were obtained for each sampling site from the Copernicus Sentinel Data (https://www.esa.int), at 50 m buffer around each sampling site and at a 10 m of spatial resolution (Table S4 in Supplementary Information). We averaged the NDVI values over the 4-month period (April–July) that coincides with the period of ant sampling ± 1 month.

We calculated the correlations among local variables and discarded variables showing a correlation coefficient > 0.70 (Table S5 in Supplementary Information)^31,52^.

As for the regional climatic variables, we extracted the AI from the global aridity database (https://cgiarcsi.community/data/global-aridity-and-pet-database)^44^. The other regional climatic variables (Table S4 in Supplementary Information), which have been shown to influence ant diversity^21,22^, were retrieved from the WorldClim database^49^ with a resolution of 30 s (~ 1 km^2^). The mean value per sampling site was extracted for each climatic variable. These variables were divided into two groups, related to temperature and precipitation respectively, and were correlated among each other within each group (Table S6 in Supplementary Information). As for local factors, climatic variables showing a correlation coefficient > 0.70 were discarded. Local environmental and regional climatic variables that were kept for further analysis are shown in Table 1.

**Table 1:** Local and climatic variables and their range at the 30 sampling sites.

Variables (unit)	Range	Variables selected
**Local**		
NDVI (unitless)	0.2–0.6	*
Dry herbaceous biomass (g/0.25m^2^)	1.2–40.1	*
Mean plant height (cm)	7.1–68.4	*
Bare soil (%)	0.0–1.0	*
Relative cover of woody plant species (%)	0.0–49.1	*
Soil N (%)	0.1–0.5	*
Soil C:N	7.6–17.2	
Soil mosses (%)	0–0.5	
Plant species	21–76	
**Climatic**		
Aridity Index (AI; unitless)	0.4–0.5	*
Annual mean temperature (bio1; °C)	15.4–17.0	*
Mean diurnal range (bio2)	9.4–11.2	*
Isothermality (bio3)	39.6–43.1	
Annual precipitation (bio12; mm)	529–604	
Precipitation of driest month (bio14)	2.6–7.4	
Precipitation seasonality (bio15)	54.7–67.5	*

Variables with a correlation coefficient > 0.70 are not included below. Variables selected as determinants of ant β-diversity after performing permutation-based forward selection (Blanchet et al.^61^) are indicated (*).

### Data analysis

To evaluate sample completeness representing ant biodiversity along the environmental gradients, we first calculated the Jackknife 1 non-parametric species richness estimator, which is used for multiple sites simultaneously^53^. Then, we measured sample completeness based on the ratio between observed species richness and the Jackknife 1 estimation.

We performed a fourth-corner analysis to assess the relationship between ant functional traits and local environmental and regional climatic variables. This technique analyses three matrices simultaneously, namely of ant species (sites by species incidence), ant functional traits (species by traits) and environmental variables (sites by environmental factors), to test the significance of all pairwise combinations of functional traits and explanatory variables^54^. The coefficient values from this analysis quantify the strength and the direction of the trait-environment relationships. To obtain the most parsimonious model, we used the *glm1path* function and the least absolute shrinkage and selection operator (LASSO) in package *mvabund* within R^55,56^.

To calculate taxonomic and functional β-diversity we first built a site per species matrix and a species per trait matrix. The site per species matrix contained presence-absence data for ant species at each site, whereas the species per trait matrix was based on mean trait value for a specific trait for a specific species. Taxonomic β-diversity was computed using the site per species matrix, while functional β-diversity was computed using the dissimilarity between species, obtained from the species per trait data, and the site per species data. The Jaccard index was used to compute β-diversity, which varies between 0 and 1. To compute taxonomic and functional β-diversity, based on pairwise dissimilarity between sites, we used function *beta* in the *BAT* package in R^56,57^. In this method the functional representation is based on functional clustering trees rather than on a functional ordination, and total species variation (Tβ_tot_; Tβ_tot_ = Tβ_repl_ + Tβ_rich_) is decomposed into variation through species replacement (Tβ_repl_) and variation due to species richness differences (Tβ_rich_). Similarly, total functional β-diversity (Fβ_tot_; Fβ_tot_ = Fβ_repl_ + Fβ_rich_) is decomposed in β replacement (Fβ_repl_), which corresponds to variation due to trait value replacement, and β richness (Fβ_rich_), which accounts for variation due to trait value loss/gain (Fβ_rich_).

Following Maire et al.^38^, to evaluate the sensibility of the results on functional β-diversity, we decided to use different sets of ant functional traits through reductions in the number of traits, combined with the original coding of the species per trait matrix or with a different trait coding. In the original trait coding, traits with a fuzzy coding only included behaviour, nesting preference, colour, mound presence and foraging strategy, instead in the different coding trait matrix, fuzzy coding was applied to all qualitative traits. We assessed mean pairwise dissimilarity for functional beta-diversity using: (a) a lower number of traits, namely 7 traits (head length, Weber’s length, diet preferences, nest preferences, behaviour, mound presence and foraging strategy) and 4 traits (Weber’s length, diet preference, behaviour and foraging strategy), and the original species per trait matrix, and (b) 11, 7 and 4 traits, with fuzzy coding applied to all qualitative traits.

To assess the influence of local environmental, regional climatic and spatial factors on taxonomic and on functional β-diversity, we conducted a variation partition procedure based on redundancy analysis^58^. The method of the variation partitioning allows us to partition β-diversity variation among local environmental, regional climatic and spatial variables as well as to assess how much of the variation in β-diversity remains unexplained^34^. To represent the spatial relationships among sites we used distance-based Moran’s eigenvector maps (dbMEM)^59^, using function *dbmem* of package *adespatial* in R^56,60^. Compared to the original principal coordinates of neighbour matrices method, identifying the eigenvectors modelling positive spatial correlation that are used in most ecological studies is easier with the dbMEM method^59^. Briefly, this analysis consists in constructing a matrix of geographic distances among sampling sites, upon which a principal coordinate analysis is performed to obtain eigenvectors that are then used as spatial explanatory variables in the variation partitioning analysis. The eigenvectors represent spatial relationships between the sites, describing wide- and small-scale variation. The first dbMEM vectors describe large scale variation, whereas later dbMEM vectors represent small scale variation.

To select the local environmental, regional climatic and spatial factors to include in the variation partition analysis, we used permutation-based forward selection^61^, to create more parsimonious sets of explanatory variables. These were selected from the group of variables from which highly correlated variables had already been excluded (local environmental and regional climatic factors included in the forward selection procedure and selected for the variation partition analysis are shown in Table 1), and from the spatial variables obtained with dbMEM. The percentage of variation in taxonomic and in functional β-diversity was explained by three sets of variables, namely local environmental, regional climatic and spatial variables and was estimated using adjusted R^2^ values^62^. The significance of each variable driving taxonomic and functional β-diversity was assessed with permutation tests^58^.

For each β-diversity component, we analyzed the total accounted for and unaccounted for variation; the variation explained individually by pure local environmental, pure regional climatic, and pure spatial effects, and by spatially structured environmental effects (shared effects between local and spatial, and between climatic and spatial variables). In some cases, the sum of pure and shared effects did not equal to the total variation explained; this can happen when an effect has a negative adjusted R^2^, and in that case, such value is presented as zero. This means that the sum is equal to the total variation explained when considering the negative value (and not considering it as zero). Negative values of adjusted R^2^ correspond to less variation being explained than by random explanatory variables and can thus be interpreted as zero^34^. We performed the variation partitioning analysis using package *vegan* in R^56,63^.

## Supplementary Information

Supplementary Information
