# Supplementary material for: Local environmental variables are key drivers of ant taxonomic and functional beta-diversity in a Mediterranean dryland
Source: Sci Rep. 2021 Jan 27;11:2292. doi: 10.1038/s41598-021-82059-w (PMC7840911; doi:10.1038/s41598-021-82059-w)
Supplement: Supplementary file 1 — Supplementary Information [file 41598_2021_82059_MOESM1_ESM.docx]

**Local environmental variables are key drivers of ant taxonomic and functional beta-diversity in a Mediterranean dryland**
Clara Frasconi Wendt, Ana Ceia-Hasse, Alice Nunes, Robin Verble, Giacomo Santini, Mário Boieiro and Cristina Branquinho

**Supplementary Information**
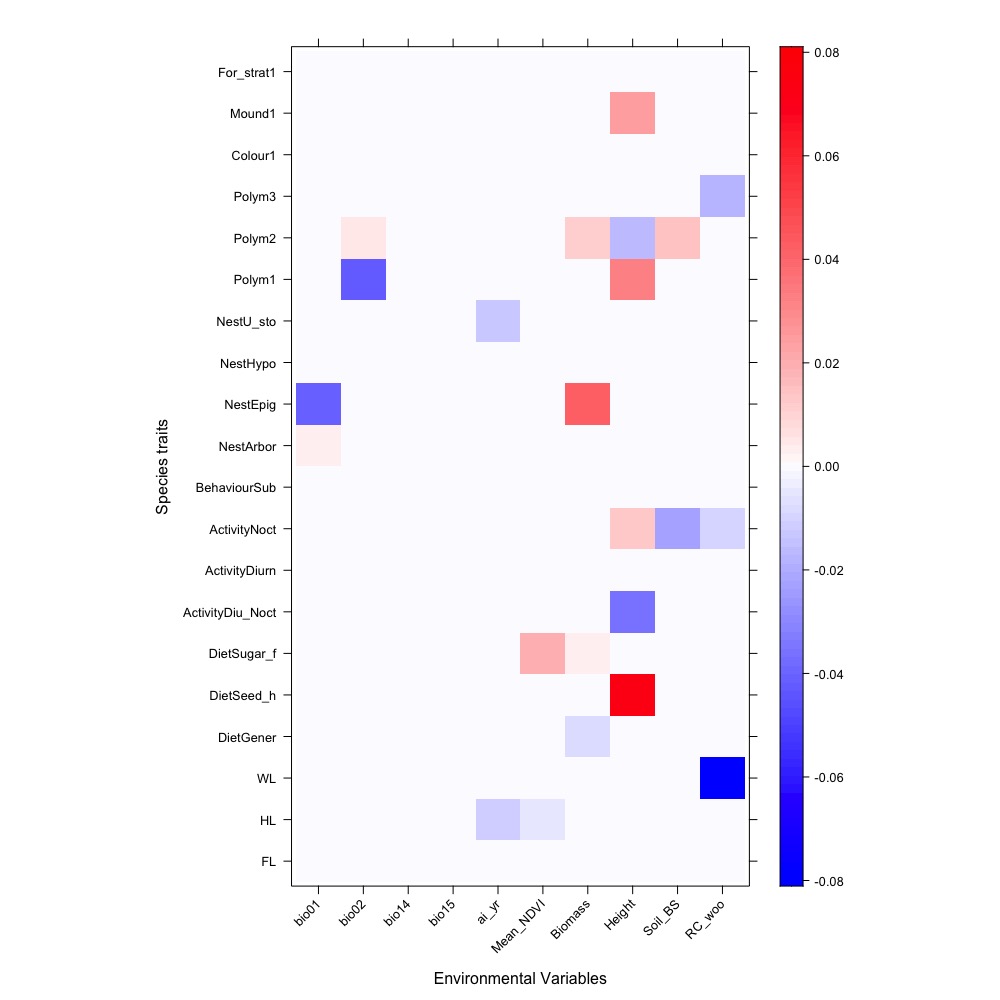


**Figure S1.** Fourth-corner plot showing the interaction coefficient between traits (y-axis) and regional climatic and local environmental variables (x-axis). Significant trait-environment associations are shown in red (positive) and in blue (negative). Different interaction strengths are shown in different colour intensities. Abbreviation ant traits: FL = femur length, HL = head length, WL = Weber’s length, DietGener = generalist diet, DietSeed_h = seed-based diet, DietSugar_f = sugar-based diet, ActivityDiu_Noct = diurnal and nocturnal activity, ActivityDiurn = strictly diurnal activity, ActovityNoct = strictly nocturnal activity, BehaviourSub = subordinate behaviour, NestArbor = arboreal nesting, NestEpig = epigeic nesting, NestHypo = hypogaeic nesting, NestU_sto = nesting under stones, Polym1 = low polymorphism, Polym2 = medium polymorphism, Polym3 = high polymorphism, Colour1 = dark-coloured, Mound1 = mound presence, For_strat1 = group foraging strategy. Abbreviations environmental variables: bio01 = Annual mean temperature, bio02 = Mean diurnal range, bio14 = Precipitation of driest month, bio15 = Precipitation seasonality, ai_yr = Aridity Index, Mean_NDVI = mean normalized difference vegetation index, Biomass = Dry herbaceous biomass, Height = mean plant height, Soil_BS = percentage of bare soil, RC_wood = relative cover of woody plant species.


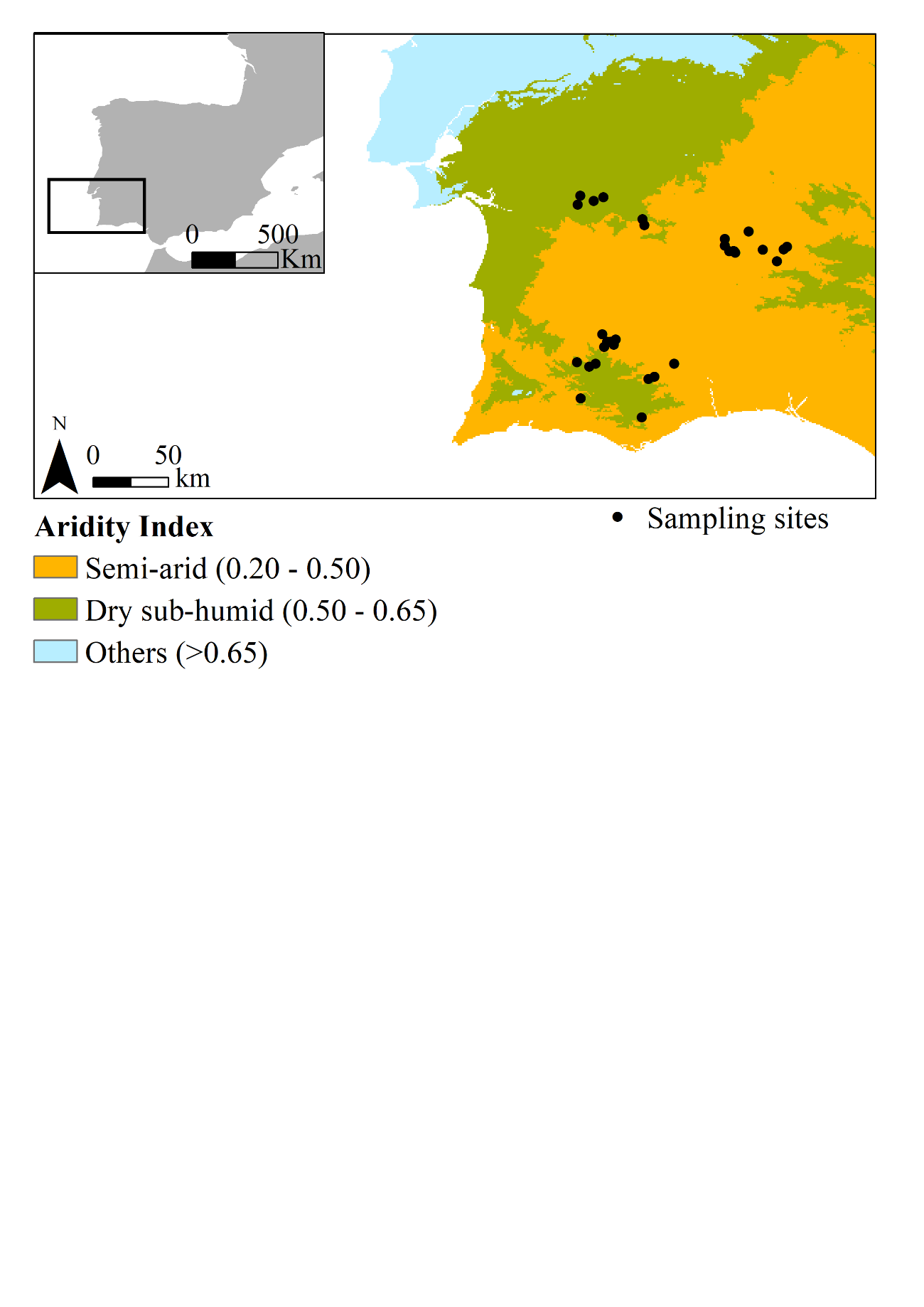


**Figure S2.** Map showing the sampling sites distribution according to aridity classes. This map was generated in ArcGIS Desktop 10.6.

**Table S1.** Ant species recorded, Aridity Index range values and number of sites where each species was observed (occupancy) along the aridity gradient (N=30). More information on ant species per site can be found here doi:10.5061/dryad.6wwpzgmwz

| **Sub-family** | **Species** | **Aridity Index range** | **Occupancy** |
| --- | --- | --- | --- |
| Dolichoderinae | *Linepithema humile* | 0.421-0.517 | 5 |
|  | *Tapinoma erraticum* | 0.528-0.531 | 2 |
|  | *Tapinoma nigerrimum* | 0.421-0.532 | 15 |
|  | *Tapinoma simrothi* | 0.420-0.532 | 20 |
| Formicinae | *Camponotus cruentatus* | 0.427-0.512 | 13 |
|  | *Camponotus foreli* | 0.430-0.512 | 5 |
|  | *Camponotus pilicornis* | 0.428-0.517 | 11 |
|  | *Camponotus sylvaticus* | 0.453-0.499 | 3 |
|  | *Cataglyphis hispanica* | 0.420-0.544 | 28 |
|  | *Cataglyphis iberica* | 0.476 | 1 |
|  | *Formica decipiens* | 0.487 | 1 |
|  | *Formica subrufa* | 0.420-0.544 | 28 |
|  | *Lasius brunneus* | 0.481-0.528 | 2 |
|  | *Lasius niger* | 0.487 | 1 |
|  | *Plagiolepis pygmaea* | 0.421-0.528 | 13 |
|  | *Plagiolepis schmitzii* | 0.428-0.532 | 10 |
| Myrmicinae | *Aphaenogaster gibbosa* | 0.420-0.544 | 15 |
|  | *Aphaenogaster iberica* | 0.420-0.517 | 7 |
|  | *Aphaenogaster senilis* | 0.428-0.544 | 23 |
|  | *Crematogaster auberti* | 0.420-0.532 | 7 |
|  | *Crematogaster scutellaris* | 0.421-0.544 | 9 |
|  | *Goniomma blanci* | 0.433-0.544 | 10 |
|  | *Goniomma hispanicum* | 0.476-0.544 | 2 |
|  | *Leptothorax acervorum* | 0.544 | 1 |
|  | *Messor barbarus* | 0.420-0.544 | 25 |
|  | *Messor capitatus* | 0.430-0.435 | 2 |
|  | *Messor lusitanicus* | 0.506-0.544 | 3 |
|  | *Pheidole pallidula* | 0.433-0.512 | 6 |
|  | *Solenopsis sp.* | 0.484 | 1 |
|  | *Temnothorax affinis* | 0.528-0.544 | 2 |
|  | *Temnothorax aveli* | 0.485-0.517 | 2 |
|  | *Temnothorax nylanderi* | 0.420-0.532 | 23 |
|  | *Temnothorax racovitzai* | 0.421-0.512 | 5 |
|  | *Temnothorax recedens* | 0.532 | 1 |
|  | *Temnothorax unifasciatus* | 0.421 | 1 |
|  | *Tetramorium forte* | 0.451-0.544 | 7 |

**Table S2**. Mean, standard deviation and variance for functional β-diversity components using: a) different numbers of ant traits with the original trait coding, and b) different numbers of ant traits with a different traits coding (fuzzy coding for all qualitative traits).

| **a)** |  |  |  |
| --- | --- | --- | --- |
| β-diversity components | Trait numbers | Mean ± standard deviation | |
| β_tot_ |  |  | |
|  | 11 traits | 0.488 ± 0.118 | |
|  | 7 traits | 0.448 ± 0.115 | |
|  | 4 traits | 0.350 ± 0.113 | |
| β_repl_ |  |  | |
|  | 11 traits | 0.317 ± 0.169 | |
|  | 7 traits | 0.284 ± 0.160 | |
|  | 4 traits | 0.200 ± 0.144 | |
| β_rich_ |  |  | |
|  | 11 traits | 0.170 ± 0.123 | |
|  | 7 traits | 0.164 ± 0.118 | |
|  | 4 traits | 0.150 ± 0.107 | |
|  |  |  | |
|  |  |  | |
| **b)** |  |  | |
| β-diversity components | Trait numbers | Mean ± standard deviation | |
| β_tot_ |  |  | |
|  | 11 traits | 0.499 ± 0.120 | |
|  | 7 traits | 0.457 ± 0.120 | |
|  | 4 traits | 0.362 ± 0.115 | |
| β_repl_ |  |  | |
|  | 11 traits | 0.330 ± 0.176 | |
|  | 7 traits | 0.290 ± 0.166 | |
|  | 4 traits | 0.210 ± 0.149 | |
| β_rich_ |  |  | |
|  | 11 traits | 0.170 ± 0.128 | |
|  | 7 traits | 0.167 ± 0.121 | |
|  | 4 traits | 0.153 ± 0.112 | |

**Table S3.** Functional traits used and their ecological meanings. References are listed in supplementary references.

| **Trait** | **Categories/Units** | **Ecological implication** |
| --- | --- | --- |
| Weber's length | cm | Ant metabolism and habitat structure (Gibb and Parr 2013) |
| Head length | cm | Diet preferences and daily foraging period (Weiser and Kaspari 2006) |
| Femur length | cm | Habitat heterogeneity, soil roughness ("size-grain" hypothesis; Kaspari and Weiser 1999) |
| Diet | generalist; seed-based; sugar-based | Resource availability and food exploitation (Arnan et al. 2012) |
| Period of activity | diurnal; nocturnal; not strictly diurnal nor nocturnal | Thermal tolerance and foraging performance (Cerdà et al. 1998; Bestelmeyer 2000) |
| Behaviour | dominant; subordinate | Determines foraging activity (Bestelmeyer 2000) |
| Nest | arboreal; epigeic; hypogeic; under stones | Environmental changes (Fagundes et al. 2015) |
| Polymorphism | absent; low; high | Colony organization of work tasks and thermal tolerance (Cerdà and Retana 2000) |
| Colour | dark; not-dark | UV-B protection, thermoregulation and foraging strategy (Frenette-Dussault et al. 2013) |
| Mound | present; absent | Habitat type and absorption/isolation of/from heat (Reymond et al. 2013) |
| Foraging strategy | individual; group | Organization of colony for food search (Traniello 1989) |

**Supplementary references**

Arnan X, Cerdá X, Retana J (2012) Distinctive life traits and distribution along environmental gradients of dominant and subordinate Mediterranean ant species. Oecologia 170:489-500.

Bestelmeyer BT (2000) The trade-off between thermal tolerance and behavioural dominance in a subtropical South American ant community. J Anim Ecol 69:998-1009.

Cerdá X, Retana J, Cros S (1998) Critical thermal limits in Mediterranean ant species: trade-off between mortality risk and foraging performance. Funct Ecol 12:45-55.

Fagundes R, Anjos DV, Carvalho R, Del-Claro K (2015) Availability of food and nesting-sites as regulatory mechanisms for the recovery of ant diversity after fire disturbance. Sociobiology 62:1-9.

Kaspari M, Weiser MD (1999) The size-grain hypothesis and interspecific scaling in ants. Funct Ecol 13:530-538.

Reymond A, Purcell J, Cherix D, Guisan A, Pellissier L (2013) Functional diversity decreases with temperature in high elevation ant fauna. Ecol Entomol 38:364-373.

Traniello JFA (1989) Foraging strategies of ants. Annu Rev Entomol 34:191-210.

Weiser MD, Kaspari M (2006) Ecological morphospace of New World ants. Ecol Entomol 31:131-142.

**Table S4.** All local and climatic variables considered prior further analysis. Abbreviations of climatic variables as those in the WorldClim database (Fick & Hijmans, 2017).

| **Variable** | **Abbreviation** | **Description** |
| --- | --- | --- |
| **Local** | Plant height | Mean plant height |
|  | Plant sp | Plant species richness (from Nunes et al. 2017) |
|  | RC woody | Relative cover woody plant species (from Nunes et al. 2017) |
|  | Biomass | Dry herbaceous biomass |
|  | NDVI | Normalized difference vegetation index |
|  | Soil LL | % leaf litter |
|  | Soil M | % mosses |
|  | Bare soil | % bare soil and rock |
|  | Soil N | Soil nitrogen content (%) |
|  | Soil C:N | Soil carbon:nitrogen ratio |
| **Climatic** | bio 1 | Annual mean temperature |
|  | bio 2 | Mean diurnal range |
|  | bio 3 | Isothermality |
|  | bio 4 | Temperature Seasonality |
|  | bio 5 | Maximum temperature of warmest month |
|  | bio 6 | Minimum temperature of coldest month |
|  | bio 7 | Temperature annual range |
|  | bio 8 | Mean temperature of wettest quarter |
|  | bio 9 | Mean temperature of driest quarter |
|  | bio 10 | Mean temperature of warmest quarter |
|  | bio 11 | Mean temperature of coldest quarter |
|  | bio 12 | Annual precipitation (mm) |
|  | bio 13 | Precipitation of wettest month |
|  | bio 14 | Precipitation of driest month |
|  | bio 15 | Precipitation seasonality |
|  | bio 16 | Precipitation of wettest quarter |
|  | bio 17 | Precipitation of driest quarter |
|  | bio 18 | Precipitation of warmest quarter |
|  | bio 19 | Precipitation of coldest quarter |
|  | AI | Ratio of mean annual precipitation to annual potential evapotranspiration |

**References**

Fick, S. E. & Hijmans, R. J. Worldclim 2: new 1-km spatial resolution climate surfaces for global land areas. *Int. J. Climatol.* **37(12),** 4302-4315 (2017).

Nunes, A. et al. Which plant traits respond to aridity? A critical step to assess functional diversity in Mediterranean drylands. *Agr. Forest Meteorol.* **239,** 176-184 (2017).

**Table S5.** Correlations between local variables (Spearman). Significantly different correlations are indicated with * (p-value < 0.05). Correlation coefficients > 0.70 were highlighted.

|  | Plant sp | RC woody | NDVI | Biomass | Plant height | Soil LL | Soil M | Soil N | Soil C:N |
| --- | --- | --- | --- | --- | --- | --- | --- | --- | --- |
| RC woody | -0.01 |  |  |  |  |  |  |  |  |
| NDVI | 0.21 | -0.21 |  |  |  |  |  |  |  |
| Biomass | 0.49* | 0.13 | 0.28 |  |  |  |  |  |  |
| Plant height | 0.28 | 0.41* | 0.05 | 0.35 |  |  |  |  |  |
| Soil LL | -0.06 | -0.36 | 0.37* | -0.02 | -0.09 |  |  |  |  |
| Soil M | 0.36* | -0.24 | 0.14 | -0.06 | -0.32 | -0.12 |  |  |  |
| Soil N | -0.06 | -0.22 | 0.53* | -0.16 | -0.2 | 0.40* | 0.13 |  |  |
| Soil C:N | 0.03 | 0 | 0.29 | -0.12 | -0.08 | 0.02 | 0.36* | 0.22 |  |
| Bare soil | -0.12 | 0.50* | -0.47* | 0.13 | 0.24 | **-0.78*** | -0.43* | -0.38* | -0.34 |

**Table S6.** Correlations between the climatic variables (Spearman); (a) Climatic variables related to temperature and (b) Climatic variables related to precipitation. Significantly different correlations are indicated with * (p-value < 0.05). Correlation coefficients > 0.70 were highlighted.

(a)

|  | bio 1 | bio 2 | bio 3 | bio 4 | bio 5 | bio 6 | bio 7 | bio 8 | bio 9 | bio 10 |
| --- | --- | --- | --- | --- | --- | --- | --- | --- | --- | --- |
| bio 2 | 0.34 |  |  |  |  |  |  |  |  |  |
| bio 3 | -0.64*** | -0.63* |  |  |  |  |  |  |  |  |
| bio 4 | 0.64*** | **0.82*** | **-0.92*** |  |  |  |  |  |  |  |
| bio 5 | 0.67*** | **0.88*** | **-0.78*** | **0.91*** |  |  |  |  |  |  |
| bio 6 | -0.03 | **-0.72*** | 0.57* | -0.58* | -0.50* |  |  |  |  |  |
| bio 7 | 0.50** | **0.93*** | **-0.78*** | **0.94*** | **0.94*** | -0.64* |  |  |  |  |
| bio 8 | 0.38* | -0.26 | 0.18 | -0.19 | -0.07 | 0.44* | -0.27 |  |  |  |
| bio 9 | **0.83***** | **0.74*** | **-0.86*** | **0.93*** | **0.93*** | -0.41* | **0.85*** | 0.08 |  |  |
| bio 10 | **0.83***** | **0.74*** | **-0.86*** | **0.93*** | **0.93*** | -0.41* | **0.85*** | 0.08 | **1.00*** |  |
| bio11 | -0.01 | **-0.76*** | 0.59* | -0.62* | -0.54* | **0.96*** | **-0.69*** | 0.57* | -0.43* | -0.43* |

(b)

|  | bio 12 | bio 13 | bio 14 | bio 15 | bio 16 | bio 17 | bio 18 |
| --- | --- | --- | --- | --- | --- | --- | --- |
| bio 13 | 0.64* |  |  |  |  |  |  |
| bio 14 | 0.28 | -0.51* |  |  |  |  |  |
| bio 15 | 0.39* | **0.91*** | -0.64* |  |  |  |  |
| bio 16 | **0.79*** | **0.96*** | -0.3 | **0.83*** |  |  |  |
| bio 17 | 0.3 | -0.47* | **0.93*** | -0.69* | -0.28 |  |  |
| bio 18 | 0.33 | -0.48* | **0.95*** | -0.67* | -0.27 | **0.95*** |  |
| bio 19 | **0.84*** | **0.86*** | -0.1 | **0.76*** | **0.94*** | -0.16 | -0.12 |
